# Enhancing Silicon Compound Heterojunction Solar Cells with Vanadium‐Doped MoO_X_ as Hole Transport Layers

**DOI:** 10.1002/advs.202505929

**Published:** 2025-05-08

**Authors:** Hongbo Cai, Xiqi Yang, Xiaofei Xu, Qinghua Zeng, Shenghou Zhou, Zilong Zheng, Dongdong Li, Yongzhe Zhang, Hui Yan

**Affiliations:** ^1^ College of Materials Science and Engineering Beijing Key Lab of Microstructure and Properties of Advanced Materials Beijing University of Technology Beijing 100124 P. R. China; ^2^ Zhangjiang Laboratory 100 Haike Road, Zhangjiang Hi‐Tech Park Shanghai 201210 P. R. China

**Keywords:** DFT calculations, finite element simulation, silicon solar cells, transition metal oxides

## Abstract

Crystalline silicon (c‐Si) solar cells dominate the global market, and the development of eco‐friendly and cost‐effective c‐Si compound solar cells with carrier‐selective passivated contacts has attracted increasing attention. This work investigated the impact of oxygen vacancies (V_O_) and vanadium (V) doping on molybdenum trioxide (MoO_X_), using a combination of first‐principles calculations and device simulations. These V_O_ defects accumulated from bulk to surface with lower energy barrier of 1.7 eV, compared to 3.4 eV on surface and 3.8 eV from surface to bulk. The surface V_O_ significantly decreased MoO_X_ work function from 6.1 eV to 4.8 eV，considering alteration in surface charges from +4 µC cm^−2^ to ‐8 µC cm^−2^. Vanadium doping increased V_O_ transport barrier by 0.1 eV, suppressing defect migration. Meanwhile, it raised work function by 0.26 eV and widened the bandgap by 0.6 eV. As hole transport layer, V‐doped MoO_X_ on illuminated side of c‐Si solar cells boosted absolute efficiency by 1.0%, compared to MoO_X_ on rear side; of this increase, 0.2% was attributed to higher work function and 0.8% was due to reduced optical losses. These findings emphasize V‐doped MoO_X_ in enhancing c‐Si compound solar cell performance and in promoting the development of efficient photovoltaic technologies.

## Graphical abstract

This work reveals that the migration and accumulation of oxygen vacancies lead to surface charge inversion in MoO_X_, which degrades device performance. Vanadium doping effectively increases the vacancy migration barrier, enhances material stability, elevates the work function, and broadens the bandgap. The study provides theoretical insights into designing high‐performance transition metal oxide interlayers for silicon compound heterojunction solar cells.

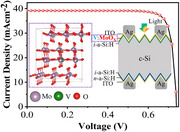

## 1 Introduction

Crystalline silicon (c‐Si) solar cells dominate ≈95%^[^ ^1^ ^]^ of the global solar cell market, known for their high efficiency^[^ ^2^ ^]^ and mature manufacturing^[^ ^3^ ^]^ processes. They are expected to maintain this leading position in the foreseeable future. Key factors impacting c‐Si cells efficiency include improvements in wafer quality, which reduce bulk recombination, as well as, advancements in passivation techniques that significantly lower surface recombination. However, recombination induced by dangling bonds on the c‐Si surface remains a critical barrier to achieving theoretical efficiency limits.

To address these challenges, novel contact schemes have been developed to provide excellent surface passivation and efficient charge transport. Carrier‐selective passivated contacts enable comprehensive passivation across the silicon surface, thereby supporting carrier transport. For instance, silicon heterojunction (SHJ) has achieved efficiencies up to 26.81%^[^ ^4^ ^]^ using intrinsic hydrogenated amorphous silicon (*i*‐a‐Si:H) and doped a‐Si:H combinations. Despite this success, SHJ requires expensive plasma‐enhanced chemical vapor deposition (PECVD) equipment and hazardous doped materials,^[^ ^5^ ^]^ and it suffers from parasitic absorption due to the small bandgap of a‐Si films.

Traditionally, the built‐in electric field of the *p*‐*n* junction in solar cells has been viewed as primary mechanism for separating photogenerated carriers. However, it was suggested that the gradient of quasi‐Fermi levels formed under illumination, can also drive carrier movement within c‐Si. Gerling et al. proposed that the energy level difference between transition metal oxides (TMOs)^[^ ^6^ ^]^ with high work functions and the c‐Si Fermi level could be considered as an alternative driving force for hole extraction.^[^ ^7^ ^]^ This selectivity can be achieved by matching the TMO work function with the conduction and valence bands of c‐Si. High work function TMOs such as molybdenum trioxide (MoO_X_)^[^ ^8^ ^]^ and vanadium pentoxide (V_2_O_X_)^[^ ^9^ ^]^ were used as hole‐selective contacts, while low work function materials such as zinc oxide (ZnO)^[^ ^10^ ^]^ and lithium fluoride (LiF_X_) were employed as electron‐selective contacts. Other high performance dopant‐free passivating contact materials used as electron transport layers have also received extensive attention, such as TiO_2_,^[^ ^11^ ^]^ TiO_X_N_Y_,^[^ ^12^ ^]^ TiN,^[^ ^6^, ^13^ ^]^ MgO_X_,^[^ ^14^ ^]^ SrF_X_,^[^ ^15^ ^]^ SiO_2_/AZO,^[^ ^16^ ^]^ and MgO/AZO^[^ ^17^ ^]^ have gained attention for their excellent carrier selectivity, passivation properties, and compatibility with low‐cost, scalable fabrication methods, making them promising candidates for advanced photovoltaic applications. Notably, the design of MgO/AZO and SiO_2_/AZO not only form a favorable energy alignment for electron transport, but also enhances interface passivation effect for device performance and stability. When integrated into the SHJ structure, MgO/AZO replaces the *n*‐a‐Si:H and achieved an impressive efficiency of 23.3%, Meanwhile, SiO_2_/AZO, when incorporated into the TOPCon structure, reached an outstanding 24.3% of efficiency. These achievements serve as an important reference for the design of future high‐efficiency silicon solar cells.

TMO‐based solar cells present many advantages, including large bandgaps, minimal parasitic absorption, and enhanced short‐circuit current density. Additionally, most TMOs can be prepared using cost‐effective, environmentally friendly methods like thermal evaporation^[^ ^8^ ^]^ or solution processing.^[^ ^18^ ^]^ Studies have demonstrated notable efficiencies, such as 19.4% achieved by using MoO_X_ and LiF_X_,^[^ ^2^ ^]^ and 22.5% by replacing the traditional *p*‐a‐Si:H emitter in SHJ cells with MoO_X_.^[^ ^19^ ^]^

MoO_X_, in particular, has garnered significant interest due to its multifunctionality, featuring a high work function (>5 eV) and a wide bandgap (∼3 eV).^[^ ^20^ ^]^ It has been widely used in organic electronic devices to reduce interface barrier energy.^[^ ^21^ ^]^ In c‐Si solar cells,^[^ ^1^, ^3^ ^]^ MoO_X_ effectively reduces interface recombination losses and enhances hole collection efficiency, thereby improving the overall photovoltaic conversion efficiency.

However, MoO_X_ is susceptible to oxygen vacancies, common defects in metal oxides^[^ ^22^ ^]^ that introduce mid‐gap states associated with Mo 4d orbitals and decrease the work function. Techniques such as ion doping^[^ ^23^ ^]^ and oxygen plasma treatment^[^ ^24^ ^]^ have been used to enhance oxygen concentration and suppress vacancy formation, thereby improving the performance of c‐Si/TMO cells.

In this work, by employing a combination of first‐principles calculations and device simulations, we explored the dynamics of suppressing V_O_ migration through doping in MoO_X_, as well as, how the doped MoO_X_ enhances the work function. Meanwhile, in device structure of MoO_X_/*i*‐a‐Si/c‐Si/*i*‐a‐Si/*n*‐a‐Si, our findings indicated that, due to the wide bandgap characteristics, illuminating from the MoO_X_ side rather than the *n*‐a‐Si side can increase the absolute device efficiency by 0.8%. Furthermore, vanadium (V) doping raised the work function by 0.26 eV, and widened the bandgap by 0.6 eV, resulting in an additional 0.2% increase in absolute efficiency. These insights are crucial for optimizing MoO_X_ in solar cells, and support the development of cost‐effective and environmentally friendly TMO‐based photovoltaic technologies.

## 2 Methodology

### 2.1 Density Functional Theory (DFT) Calculations

First‐principles calculations were performed using the Vienna Ab initio Simulation Package (VASP)^[^ ^25^ ^]^ based on density functional theory (DFT).^[^ ^26^ ^]^ The projector augmented wave (PAW) method with a plane wave basis set and the standard Perdew–Burke–Ernzerhof (PBE)^[^ ^27^ ^]^ exchange‐correlation functional were used for initial structural optimizations and transition state calculations. Electronic structure properties and total energy of the materials were calculated using the HSE06 hybrid functional.^[^ ^28^ ^]^ Van der Waals corrections were applied using Grimme's DFT‐D3^[^ ^29^ ^]^ method to describe the weak interactions within the system.

All structural optimizations were conducted using the conjugate gradient (CG) method, with a kinetic energy cutoff of 500 eV, an energy convergence threshold of 10^−5^ eV, and a force threshold of 0.01 eV Å^−1^. The Brillouin zone integrations utilized a Gamma‐centered Monkhorst‐Pack K‐point grid,^[^ ^30^ ^]^ with a 3 × 2 × 3 grid for bulk models and a 3 × 1 × 3 grid for slab models. Calculations involving defects and transition states were performed using a supercell structure containing 144 atoms. The formation energy of oxygen vacancy defects was calculated using the formula in Equation (1),^[^ ^31^ ^]^ where the chemical potential of oxygen was taken as half the energy of O_2_, as reported in literature.^[^ ^32^ ^]^

(1) $$E_{f}=E_{defect}-E_{perfect}+\Sigma_{n_{i}}\mu_{i}$$

where *E_defect_* is the total energy of the defect‐containing system consisting of *n_i_* atoms, with atomic chemical potential µ_*i*_. From this is subtracted the total energy of the perfect stoichiometric cell, *E_perfect_*, with the sum over the removed species for mass balance.

The transition state calculations were performed using the climbing image nudged elastic band (CI‐NEB)^[^ ^33^ ^]^ method, inserting several intermediate configurations between initial and final states and optimizing them by applying spring forces until a predefined convergence criterion was met. At least five intermediate configurations were inserted, with a force convergence criterion set to 0.05 eV Å^−1^.

### 2.2 Device Simulations

Finite element simulation of c‐Si solar cells with MoO_X_ hole transport layers (HTL) were conducted using Silvaco TCAD software. The simulations were based on mathematical models, including the Poisson equation, carrier continuity equations, and drift‐diffusion equations, iteratively solved until self‐consistency was achieved. All simulation parameters were listed in Tables S1–S3 and Figure S3 (Supporting Information), sourced from previous literature^[^ ^4^, ^34^ ^]^ and the Silvaco database. Refractive index and absorption coefficients for c‐Si, a‐Si:H, transparent electrode indium tin oxides (ITO), and Ag were extracted from the Silvaco database, while those for MoO_X_ were obtained from literature.^[^ ^35^ ^]^

Simulations were performed at room temperature (300K) and employed Auger recombination models, Shockley‐Read‐Hall (SRH) recombination models, band‐to‐band (B2B) tunneling models, direct tunneling models, and thermal emission models. By modeling the current‐voltage characteristics under AM1.5G spectrum illumination, which represented the standard solar spectrum, we analyzed the electrical behavior and performance of the solar cells.

## 3 Results and Discussion

### 3.1 Structural and Electronic Properties of MoO_3_

**Figure 1:**
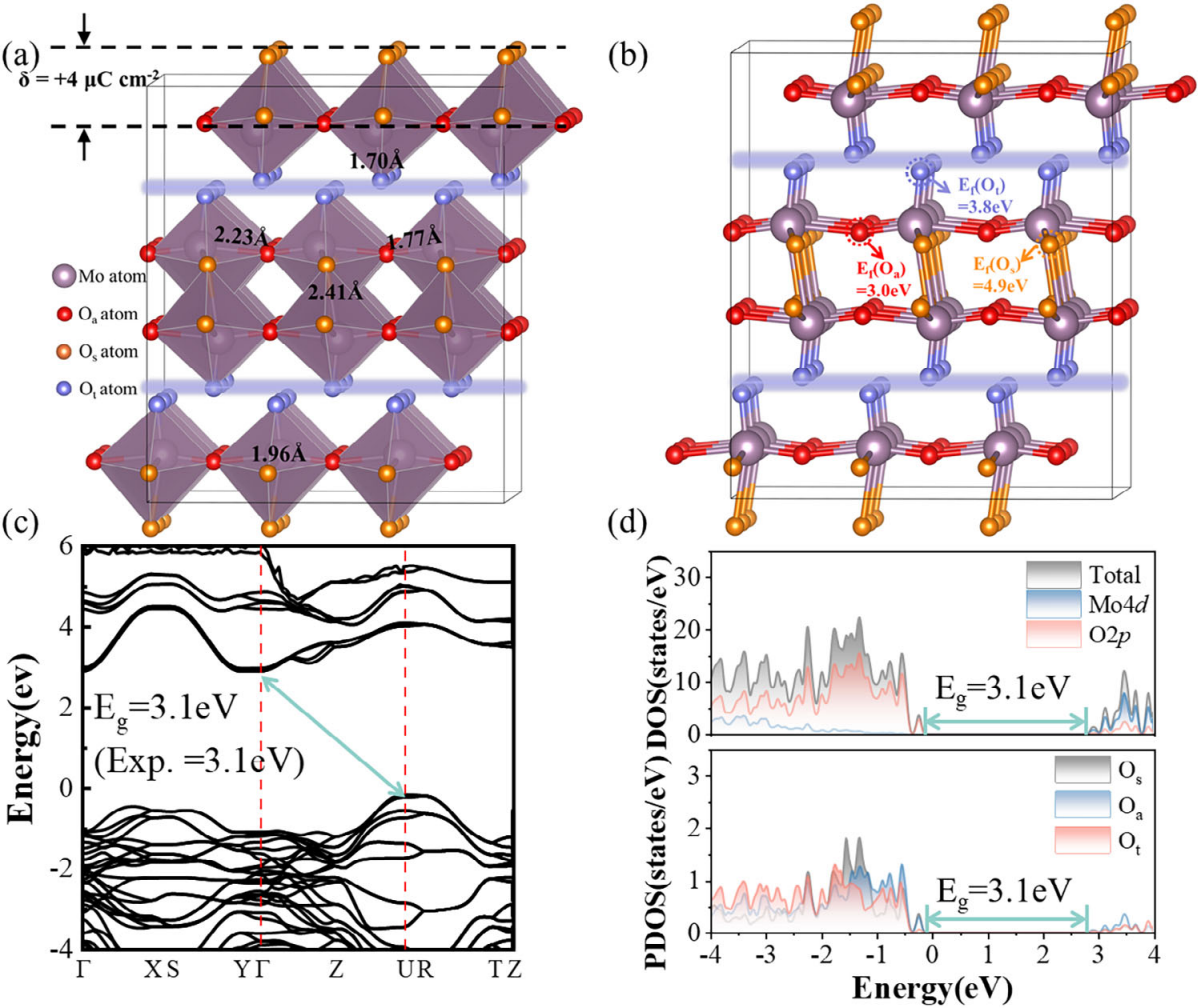
a) Structure and surface charge δ of bulk MoO_3_ with various types of oxygen atoms, including terminal oxygen (O_t_), symmetrical oxygen (O_s_), and asymmetrical oxygen (O_a_), with blue, orange, and red color, respectively. b) Formation energies (E_f_) of V_O_ defects at terminal oxygen, symmetrical oxygen, and asymmetrical oxygen sites. c) Band structure of pristine MoO_3_. d) Total and partial density of states (DOS/PDOS) of pristine MoO_3_.

**Figure 2:**
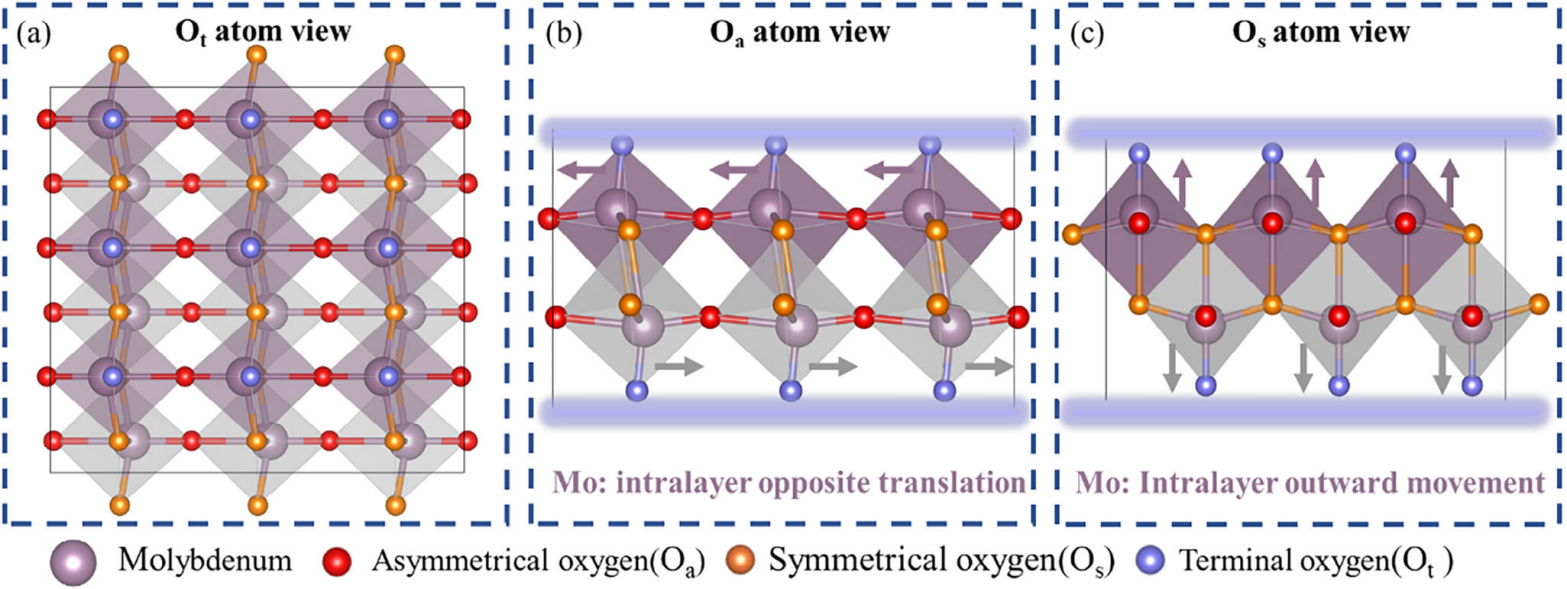
The distorted MoO_3_ octahedron structure from the perspective of a) Ot atoms, b) O_a_ atom, and c) O_s_ atom, respectively.

Bulk MoO_3_ exhibits a stable orthorhombic phase with MoO_6_ octahedron layered structure, which share edges and corners along the [010] direction. This arrangement includes terminal oxygen (O_t_), symmetrical oxygen (O_s_), and asymmetrical oxygen (O_a_), see **Figure** **1**a. The bond lengths were obtained at 1.70 Å for Mo‐O_t_, 1.96Å and 2.41 Å for Mo‐O_s_, and 1.77 Å and 2.23 Å for Mo‐O_a_. Each nanosheet of MoO_3_ consists of two layers of MoO_6_ octahedra, and all the MoO_6_ octahedra were distorted; while in the adjacent layers of MoO_6_ octahedra, the center Mo atoms were translated in opposite directions, and the O_t_ atoms made intralayer outward movements as well, see **Figure** **2**. Based on DFT calculations, the energy of this distorted octahedral arrangement was 3.3 eV lower than that of undistorted MoO_6_ octahedron, which led to the inconsistency in bond lengths for Mo‐O_s_ and Mo‐O_a_. Meanwhile, the bond angles in distorted MoO_6_ octahedra were as follows: O_t_‐Mo‐O_a_ angles were 90.3° and 102.7°, O_s_‐Mo‐O_a_ angles were 91.1° and 75.9°, O_t_‐Mo‐O_s_ angle was 104.9°, as well as, O_s_‐Mo‐O_s_ angle was 72.7°, see Figure S1 (Supporting Information). The distorted octahedron in other transition metal oxides were observed as well, for instance, V_2_O_5_^[^ ^36^ ^]^ and WO_3_.^[^ ^37^ ^]^ The MoO_3_ equilibrium structure was obtained with HSE06 hybrid functional, yielding lattice constants in good agreement with experimental data, see **Table** **1**.

**Table 1:** Lattice constants in bulk MoO_3_ structure.

	This work [Å]	Experimental [Å]
a	3.92	3.96^[^ ^38^ ^]^
b	14.01	13.86^[^ ^38^ ^]^
c	3.70	3.70^[^ ^38^ ^]^

In order to investigate electronic properties, the total density of states (TDOS) and band structure of pristine MoO_3_ were calculated using the same functionals, see **Figure** 1c,d. An indirect bandgap of 3.1 eV was obtained, consistent with the experimental finding (3.1 eV).^[^ ^39^ ^]^ The valence band maximum (VBM) and conduction band minimum (CBM) were located at the U (0.5, 0.0, 0.5) and Г (0.0, 0.0, 0.0) points, respectively. Figure 1d The TDOS presented that the VBM was contributed by the 2p orbitals of O_s_ and O_a_ atoms, while the CBM was mainly derived from Mo 4d orbitals with minor contributions from O_a_ 2p orbitals.

### 3.2 Oxygen Vacancy Migration Mechanisms in MoO_X_

**Figure 3:**
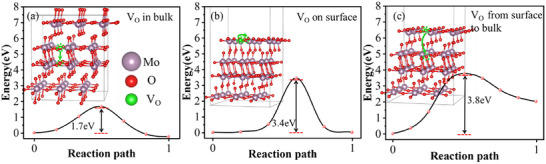
Potential energy barriers of oxygen vacancies along with three transport pathways, a) within the bulk, b) on the surface, and c) surface‐to‐bulk transports.

MoO_X_ are prone to reduction reactions, where Mo atoms are reduced from a +6 to the +5 oxidation state,^[^ ^39^, ^40^ ^]^ leading to oxygen deficiencies and the formation of V_O_. These defects can lower MoO_X_ work function, adversely affecting solar cell performance. Then, we estimated the formation energy (E_f_) of V_O_ defects through thermodynamic Equation (1), finding E_f_ values of 3.8, 3.0, and 4.9 eV, for O_t_, O_a_, and O_s_ sites, respectively. This indicated O_a_ sites as the most energetically favorable for V_O_ defects. Therefore, we focused on the migration mechanisms of V_O_ defects at O_a_ site, exploring three primary pathways: bulk transport, surface transport, and surface‐to‐bulk transport, see **Figure** **3**. Bulk transport exhibited the lowest energy barrier of 1.7 eV, while surface and surface‐to‐bulk transport presented higher potential barriers of 3.4 and 3.8 eV, respectively. Therefore, V_O_ defects tend to accumulate on the surface as they migrate from the bulk, highlighting the necessity to address surface accumulation to improve MoO_X_ stability.

**Figure 4:**
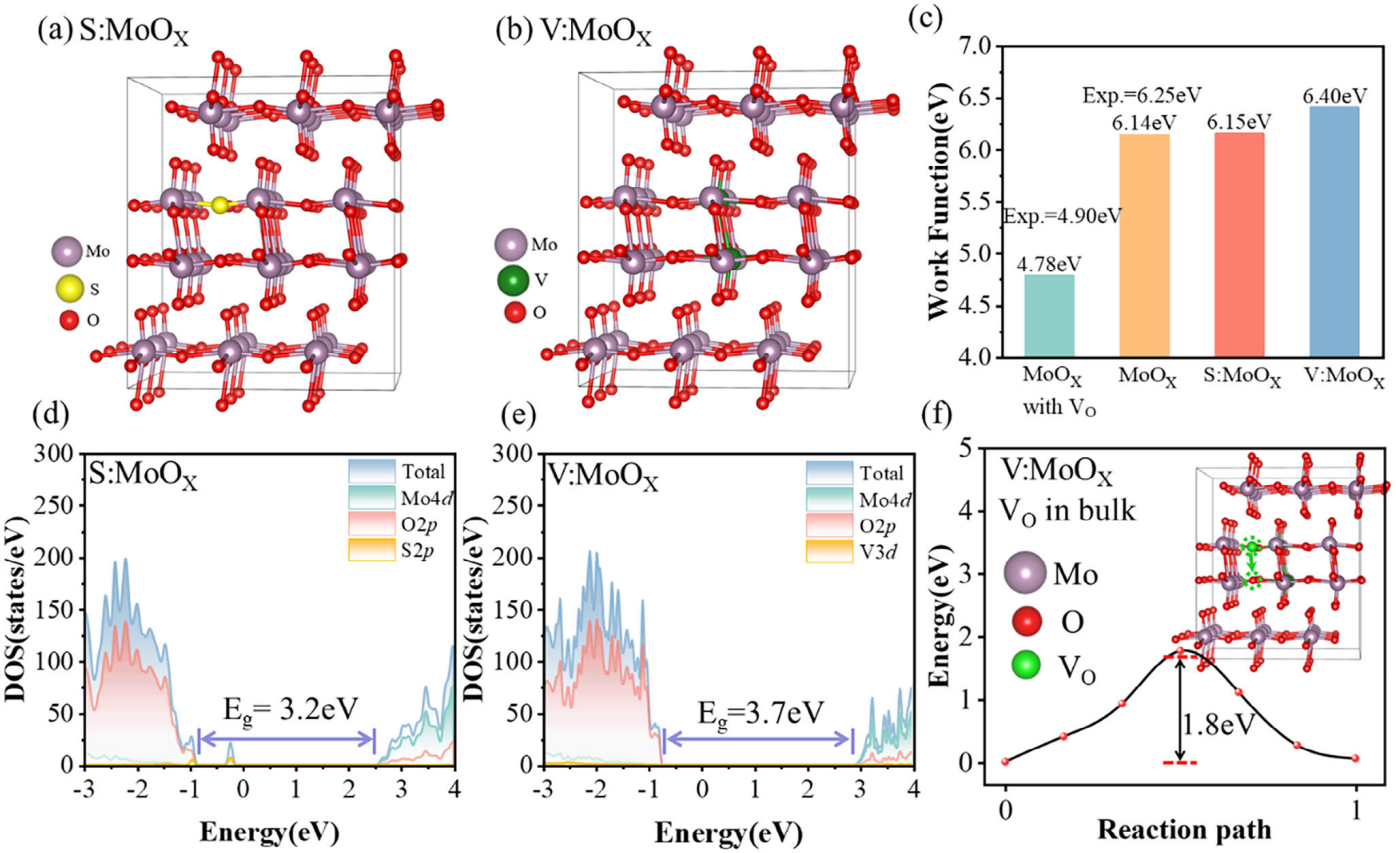
Structure of a) S‐doped MoO_X_ and b) V‐doped MoO_X_. c) Work function of MoO_X_ with oxygen vacancy, pristine MoO_X_, S‐doped MoO_X_, and V‐doped MoO_X_. Total and partial density of states (DOS/PDOS) of d) S‐doped MoO_X_ and e) V‐doped MoO_X_. f) Potential energy barriers of oxygen vacancy migration pathways in V‐doped MoO_X_.

The work functions (WF) of pristine MoO_X_ and MoO_X_ with oxygen vacancies (V_O_:MoO_X_) were obtained, which were 6.14 eV (experimental WF = 6.25 eV^[^ ^41^ ^]^), 4.78 eV (experimental WF = 4.90 eV^[^ ^42^ ^]^), respectively, and V_O_ notably decreased the WF by 1.36 eV see **Figure** **4**c. High WF in pristine MoO_X_ was due to the positive surface charges density (δ = 4 µC cm^−2^), see Figure 1a, which increases the Coulomb attraction that electrons have to overcome when escaping from the material surface. However, the surface V_O_ defects altered the MoO_X_ surface charges to negative (δ = ‐8 µC cm^−2^), which facilitated the escape of electrons and decreased the work function of V_O_:MoO_X_.

### 3.3 Impact of Vanadium Doping on MoO_X_

Sulfur (S) has the same outermost electron configuration as oxygen (O), however, binds these electrons less tightly, making S substitution equivalent to *n*‐type doping. Vanadium (V), having one fewer electron in its outer shell compared to molybdenum (Mo), acts as a *p*‐type dopant. Replacing Mo with V is equivalent to *p*‐type doping. Therefore, we introduced S and V as dopants to investigate the properties of doped MoO_X_. Furthermore, the optimal vanadium doping concentration was determined to be 5%, see Figure S2 (Supporting Information). Both S‐doped and V‐doped MoO_X_ structures are shown in Figure 4. Subsequently, the work functions of S‐doped and V‐doped MoO_X_ were compared, which were 6.15 and 6.40 eV, respectively. While, compared to 6.14 eV of WF MoO_X_, sulfur doping caused a slight (0.01 eV) WF increase, and vanadium doping significantly enhanced the WF by 0.26 eV, see Figure 4.

We further investigated the electronic structures of the doped systems. S‐doped MoO_X_ displayed a bandgap of 3.2 eV, with VBM contributions mainly from O 2p orbitals and CBM contributions from Mo 4d orbitals. A defect state appeared 0.4 eV above the VBM, involving contributions from O 2p and S 2p orbitals. In contrast, V‐doped MoO_X_ showed an increased bandgap of 3.7 eV, attributed to a decreased VBM, thus widening the gap. Its VBM and CBM were similarly contributed by O 2p and Mo 4d orbitals, respectively, without introducing defect states. Therefore, we investigated V_O_ migration in V‐doped MoO_X_, see Figure 4, noting an increased migration barrier of 1.8 eV compared to 1.7 eV in the undoped system. This suggests that V doping may inhibit V_O_ migration, enhancing the MoO_X_ stability.

### 3.4 Photovoltaic Characterization of Vanadium Doping MoO_X_ Solar Cells

**Figure 5:**
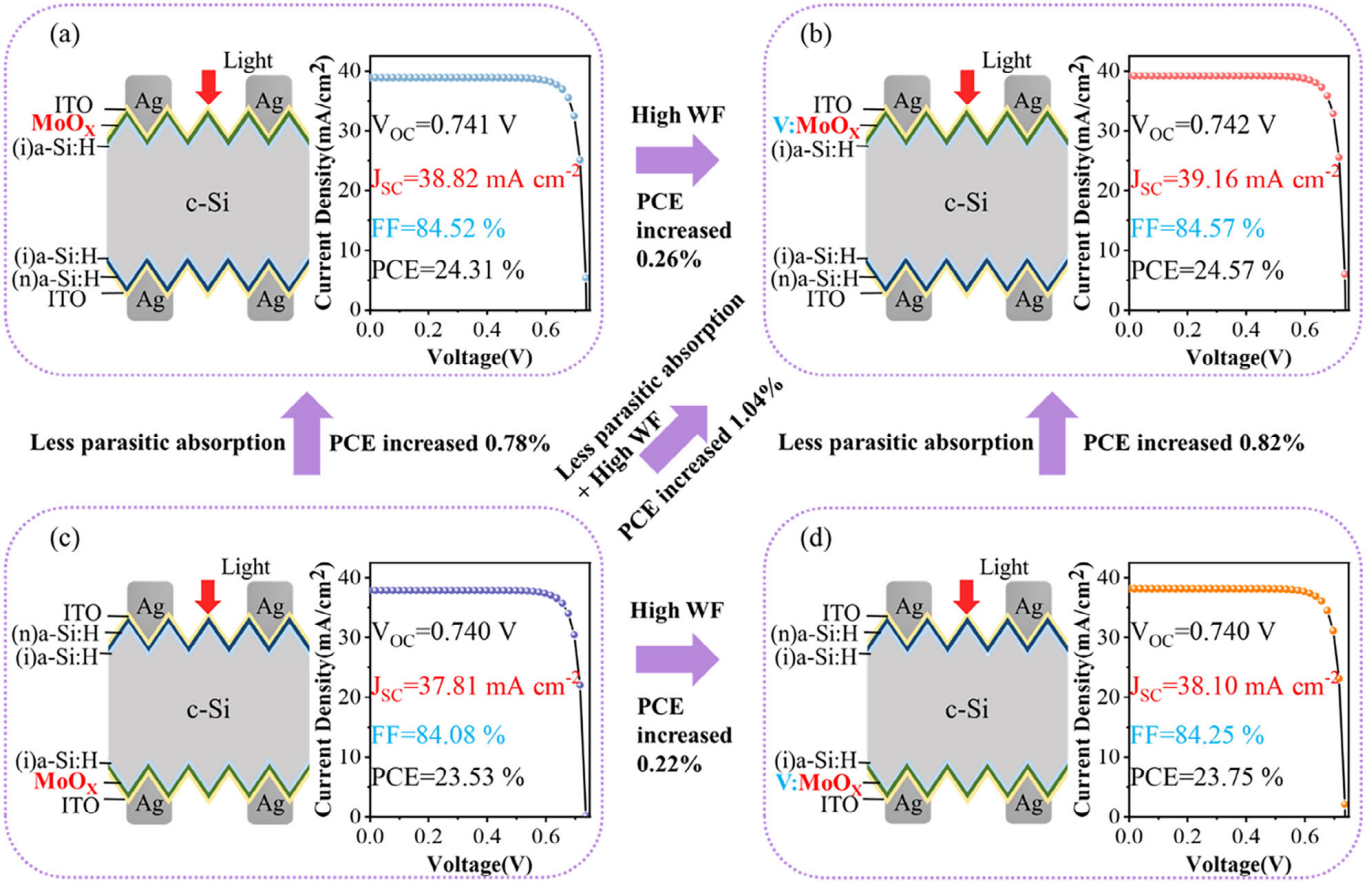
Device structures and current–voltage curves of silicon compound solar cells with hole transport layer of a) MoO_X_ on the illuminated side, b) V‐doped MoO_X_ on the illuminated side, c) MoO_X_ on the rear side, and d) V‐doped MoO_X_ on the rear side, respectively.

The above first‐principles calculations indicated V doping may enhance the performance of MoO_X_. To further confirm the effects, the photovoltaic properties of V‐doped MoO_X_ (V:MoO_X_) were investigated by using Silvaco TCAD device simulations. Our study focused on four c‐Si solar cell device structures, employing MoO_X_ and V:MoO_X_ as hole transport layers (HTL) on both the illuminated side and rear side. Moreover, the HTL in this study was fabricated by thermal evaporation, with detailed experimental procedures provided in the Supporting Information. The optimal thickness of V:MoO_X_ as the HTL was determined to be 4 nm, as shown in Figure S4 (Supporting Information). The device structure consisted of Ag/ITO/HTL/*i*‐a‐Si:H/c‐Si/*i*‐a‐Si:H/*n*‐a‐Si:H/ITO/Ag, see **Figure** **5**.

When pristine MoO_X_ was used on the rear side, it achieved a PCE of 23.53% with short‐circuit current density (J_SC_) of 37.81 mA cm^−2^, open‐circuit voltage (V_OC_) of 0.740 V, and the fill factor (FF) of 84.08%, see Figure 5c. When MoO_X_ was placed on the illuminated side, the PCE increased to 24.31%, J_SC_ rose to 38.82 mA cm^−2^, and FF improved to 84.52%, marking a 0.78% efficiency increase compared to when MoO_X_ was on the rear side. This improvement was attributed to the MoO_X_ wide bandgap of 3.1 eV, much larger than 1.74 eV^[^ ^43^ ^]^ of *n*‐a‐Si:H, and the former significantly reduced parasitic absorption and enhanced spectral absorption, thereby minimizing optical losses and boosting overall cell performance. Correspondingly, the external quantum efficiency (EQE) exhibited improved light response in the short‐wavelength region, as shown in Figure S5 (Supporting Information).

The device with V:MoO_X_ on the rear side yielded a PCE of 23.75%, with J_SC_ of 38.10 mA cm^−2^, V_OC_ of 0.740 V, and FF of 84.25%, see Figure 5d. V‐doped MoO_X_ enhanced the efficiency by 0.22%, attributed to the increased work function from 6.14 to 6.40 eV with V doping. When the V:MoO_X_ layer was utilized on the illuminated side, the PCE increased to 24.57%, with J_SC_ of 39.16 mA cm^−2^ and FF of 84.57%. Compared to V:MoO_X_ on the rear side, the PCE saw an increase of 0.82%, in consideration of V:MoO_X_ wide bandgap significantly reduced parasitic absorption and optical losses. Finally, the comparative results indicated that the c‐Si solar cell with V:MoO_X_ as the HTL on the illuminated side enhanced the efficiency by 1.04% compared to MoO_X_ on the rear side; while, 0.2% increase come from high WF of V‐doped MoO_X_, and the other 0.8% increase contributed to low optical losses of V‐doped MoO_X_.

Meanwhile, we fabricated silicon compound heterojunction solar cells using V‐doped MoO_X_ and pure MoO_X_ as HTLs on the rear side for validation. The experimental results demonstrated the impact of V‐doping MoO_X_, with the efficiency increasing by 0.4% compared to pure MoO_X_ HTL device, see Table S4 and Figure S7 (Supporting Information). This improvement aligned with the theoretically predicted enhancement of 0.2%. The experimental outcomes confirmed the viability of V:MoO_X_ HTLs for silicon compound heterojunction solar cells, underscoring their potential for enhancing photovoltaic performance.

## 4 Conclusion

In summary, by combining first‐principles calculations with device simulations, we investigated the impact of oxygen vacancies (V_O_) and vanadium (V) doping on the electronic properties of MoO_X_. The V_O_ defects exhibited energetic stability at asymmetrical oxygen sites, with a low formation energy of 3.0 eV, compared to higher energies at terminal (3.8 eV) and symmetrical (4.9 eV) oxygen sites. Then, V_O_ defects migration presented the lowest energy barrier of 1.7 eV within the bulk, compared to 3.4 and 3.8 eV on the surface and surface‐to‐bulk transport, respectively, indicating a tendency for surface accumulation as V_O_ migrate from the bulk. This migration significantly reduces the work function of MoO_X_ from 6.14 to 4.78 eV, which is consistent with experimental values of 6.25 and 4.90 eV. This is because the MoO_X_ surface charges alteration from positive 4 µC cm^−2^ (without V_O_) to negative 8 µC cm^−2^ (with V_O_), and the negative charges facilitated the escape of electrons.

V doping increased transport barrier for V_O_ defects by 0.1 eV, reducing V_O_ migration and enhancing MoO_X_ stability. Additionally, V doping elevated the work function by 0.26 eV and widened bandgap by 0.6 eV, improving hole selectivity and reducing optical losses in c‐Si solar cells. When V‐doped MoO_X_ was used as hole transport layer on the illuminated side of c‐Si solar cells, it enhanced absolute efficiency by 1.0% compared to MoO_X_ on the rear side, with 0.2% attributed to the higher work function and 0.8% due to reduced optical losses. These findings highlighted the potential of V‐doped MoO_X_ to enhance the efficiency of c‐Si solar cells and support the development of high‐efficiency, environmentally friendly silicon compound photovoltaic technologies.

## Conflict of Interest

The authors declare no conflict of interest.

## Supporting information



Supporting Information

## Data Availability

The data that support the findings of this study are available from the corresponding author upon reasonable request.
