# Supplementary material for: Enhancing Silicon Compound Heterojunction Solar Cells with Vanadium‐Doped MoOX as Hole Transport Layers
Source: Adv Sci (Weinh). 2025 May 8;12(28):2505929. doi: 10.1002/advs.202505929 (PMC12302542; doi:10.1002/advs.202505929)
Supplement: Supplementary file 1 — Supporting Information [file ADVS-12-2505929-s001.docx]

# Supporting Information

**Enhancing Silicon Compound Heterojunction Solar Cells with Vanadium-Doped MoO_X_ as Hole Transport Layers**

Hongbo Cai^1,#^, Xiqi Yang^1,#^, Xiaofei Xu^1,#^, Qinghua Zeng^1^, Shenghou Zhou^1^, Zilong Zheng^1,^*, Dongdong Li^2,^*, Yongzhe Zhang^1,^* and Hui Yan^1^

^1^ *College of Materials Science and Engineering, Faculty of Information Technology, Beijing University of Technology, China, Beijing, 100124, P. R. China.*

^2^ *Zhangjiang Laboratory, 100 Haike Road, Zhangjiang Hi-Tech Park, Shanghai 201210, P. R. China*

^#^ These authors contributed equally to this paper.

Corresponding author E-mail:

zilong.zheng@bjut.edu.cn; lidd@zjlab.ac.cn; yzzhang@bjut.edu.cn

To further confirm the effects, the photovoltaic properties of V-doped MoO_X_ (V:MoO_X_) was investigated by using Silvaco TCAD device simulations. Our study focused on four c-Si solar cell device structures, employing MoO_X_ and V:MoO_X_ as hole transport layers (HTL) on both the illuminated side and rear side. The devices are based on the SHJ heterojunction structure, consisting by Ag/ITO/HTL/*i*-a-Si:H/c-Si/*i*-a-Si:H/*n*-a-Si:H/ITO/Ag. The parameters required for simulating each layer structure are listed in Table S1.

**Table S1.** Band gap (E_g_), electronic affinity (EA), density of states in conduction band (N_C_) and valence band (N_V_), donor density (N_D_), acceptor density (N_A_), dielectric constant (ε), as well as mobilities of electron (μ_n_) and hole (μ_p_) for c-Si, (n)a-Si:H, (p)a-Si:H, (i)a-Si:H and MoO_X_ used in device simulations.

|  | Si | (n)a-Si:H | (p)a-Si:H | (i)a-Si:H | MoO_X_ | V:MoO_X_ |
| --- | --- | --- | --- | --- | --- | --- |
| E_g_ (eV) | 1.12 | 1.7 | 1.7 | 1.7 | 3.1^a^ | 3.7 ^a^ |
| Thickness (nm) | 1.3×10^5^ | 15 | 20 | 5 | 4^[1]^ | 4 |
| EA (eV) | 4.10 | 3.8 | 3.8 | 3.8 | 6.14^a^ | 6.4 ^a^ |
| ε | 11.9 | 11.9 | 11.9 | 11.9 | 5.9^[2]^ | 5.9^[2]^ |
| N_C_ **(**cm^−3^**)** | 2.8×10^18^ | 2.0×10^20^ | 2.0×10^20^ | 2.0×10^20^ | 2.85×10^19^  ^[2]^ | 1.22×10^19a^ |
| N_V_ **(**cm^−3^**)** | 1.0×10^19^ | 2.0×10^19^ | 2.0×10^20^ | 2.0×10^20^ | 2.68×10^19^  ^[2]^ | 2.24×10^19a^ |
| N_D_ **(**cm^−3^**)** | 3.0×10^15^ | 1.0×10^19^ | \ | \ | \ | \ |
| N_A_ **(**cm^−3^**)** | \ | \ | 1.0×10^19^ | \ | 1×10^19[2]^ | 2.37×10^19a^ |
| μ_n_ **(**cm^2^V^−1^s^−1^**)** | 1350 | 25 | 25 | 20 | 1107^[2]^ | 173.46^a^ |
| μ_p_ **(**cm^2^V^−1^s^−1^**)** | 450 | 5 | 5 | 4 | 424.6^[2]^ | 74.95^a^ |

^a^ obtained from the DFT calculations in this work

The defect state parameters at the interface between crystalline silicon and amorphous silicon^[3]^, as well as the defect parameters at the interface between amorphous silicon and MoOx^[4]^, are presented. These parameters are derived from published studies.

**Table S2.** Parameters of defect states on c-Si/MoO_X_ interface used in device simulations^[3]^.

| Parameter | *i*-a-Si:H |
| --- | --- |
| Tunneling mass | 0.1 |
| Urbach energy (VB tail) (meV) | 50 |
| Urbach energy (CB tail) (meV) | 35 |
| Urbach tail pre-factor (cm^-3^eV^-1^) | 1.88 × 10^21^ |
| Urbach tail e/h capture cross section (cm^2^) | 7 × 10^-16^ |
| Gaussian donor peak position (eV) | 0.89 |
| Gaussian acceptor peak position (eV) | 1.09 |

**Table S3.** Parameters of defect states on MoO_X_/*i*-a-Si:H interface^[4]^.

| Parameter | MoO_X_ |
| --- | --- |
| Defect type | Neutral |
| Capture cross section electrons (cm^2^) | 10^−19^ |
| Capture cross-section holes (cm^2^) | 10^−19^ |
| Energy with respect to Reference (eV) | 0.06 |
| Total defect density (cm^-2^) | 10^10^ |

Considering layered arrangement of the crystal structure and the variation in Mo-O bond lengths, the MoO_6_ is not in an ideal octahedron configurations but showed significant distortion. This reduces the system symmetry, breaking the degeneracy of the t_2g_ and eg orbitals^[5]^ . As a result, the interaction between Mo and O atoms in different directions with the t_2g_ sub-orbitals, causing the t_2g_ orbitals to split in energy^[5]^. The d_z_^2^ orbital is significantly influenced by the Mo-O_s_ bond, resulting in an increase in energy, while the d_x_^2^_-y_^2^ orbital showed minimal change, as shown in Figure S1 (c). In the projected density of states (PDOS), this energy splitting broadened the t_2g_ and e_g_ states, and resulted in the appearance of multiple peaks^[6]^ within the same region, as illustrated in Figure S1 (d). The distortion of the octahedron may introduce new electronic states, increasing the density of states in the low-energy region (near the Fermi level), which manifests as multiple peaks. This is attributed to the splitting of the t_2g_ states. The uneven Mo-O bond lengths cause the d_xy_, d_xz_, and d_yz_ orbitals to lose degeneracy. Among them, the d_xz_ and d_yz_ orbitals are more significantly influenced by the Mo-O_s_ bond, resulting in slightly higher energy than the d_xy_ orbital, leading to the appearance of multiple peaks in the low-energy region. The high-energy region corresponds to the e_g_ states (d_x_^2^_-y_^2^ and d_z_^2^). The distorted octahedron makes the d_z_^2^ orbit significantly affected by the extension of the xy axial bond, resulting in an energy increase. In the PDOS, this may appear as a peak shift toward higher energy in the high-energy region, while the d_x_^2^_-y_^2^ orbital contribution may manifest as further broadening. The rearrangement of electronic states caused by the distortion may also alter the intensity distribution of the density of states for different orbitals. The rearrangement of electronic states due to the distortion may also change the intensity distribution of the DOS for different orbitals.

**
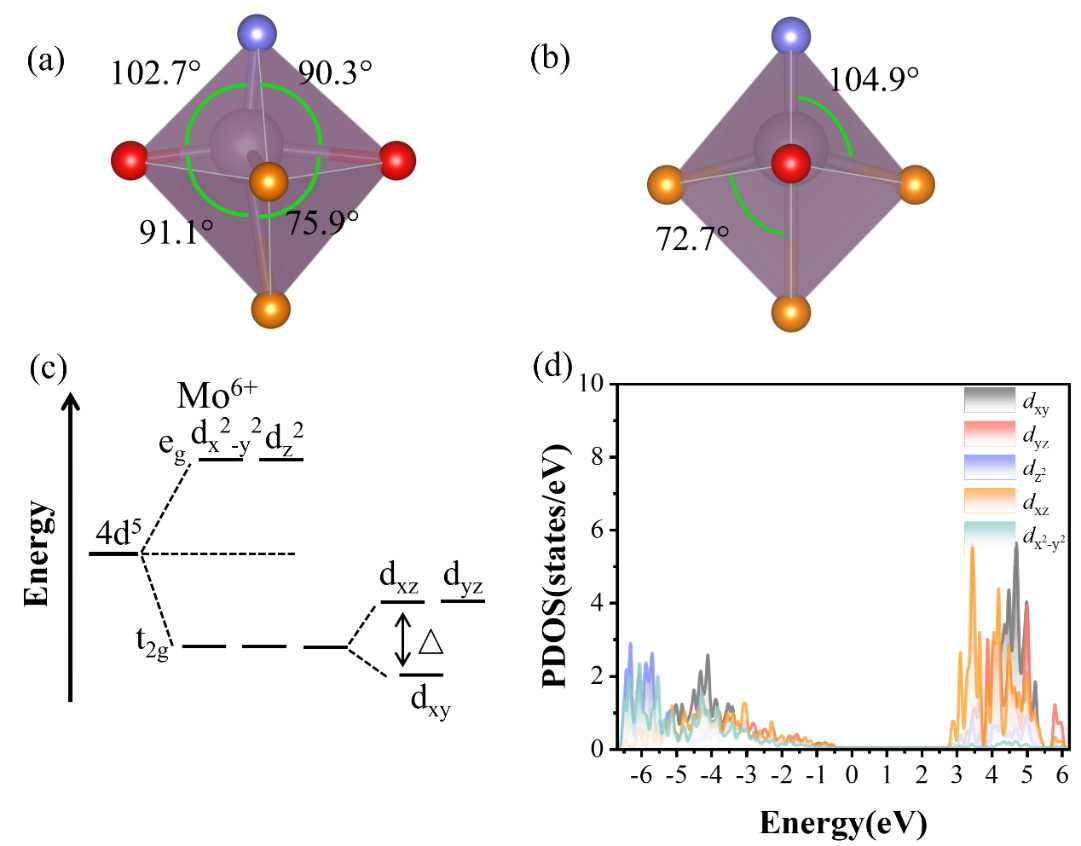
**

**Figure S1.** MoO_6_ octahedra from (a) side view of O_s_ and (b) side view of O_a_, respectively. (c) The energy levels of Mo^6+^ 4d orbital, (d) projected density of states (PDOS) of Mo element 4d orbitals.

We systematically investigated the impact of vanadium doping concentration on the electronic properties and device performance of MoO_X_. A 5% doping level was identified as optimal, achieving a maximum bandgap of 3.7 eV and enhanced work function, thereby improving light absorption and charge extraction. Device simulations confirmed peak efficiency of 24.57% at this concentration, balancing electronic modulation and optical performance. Therefore, 5% is determined to be the optimal vanadium doping level.

**
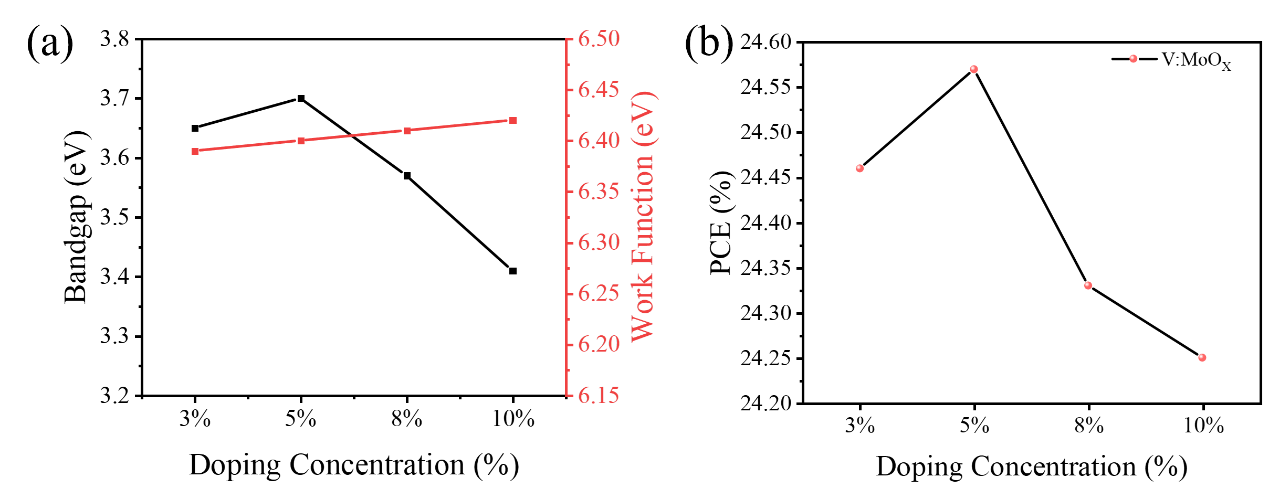
**

**Figure S2.** (a) Bandgap, work function, and (b) efficiency of V:MoO_X_ with different V-doping concentrations.

The refractive index for pure MoO_X_, and we employed the same data for V-doped MoO_X_ in device simulations, considering the low concentration V doping in MoO_X_. Meanwhile the refractive index of both MoO_X_ and VOx are almost the same as each other, as shown in Figure S3.


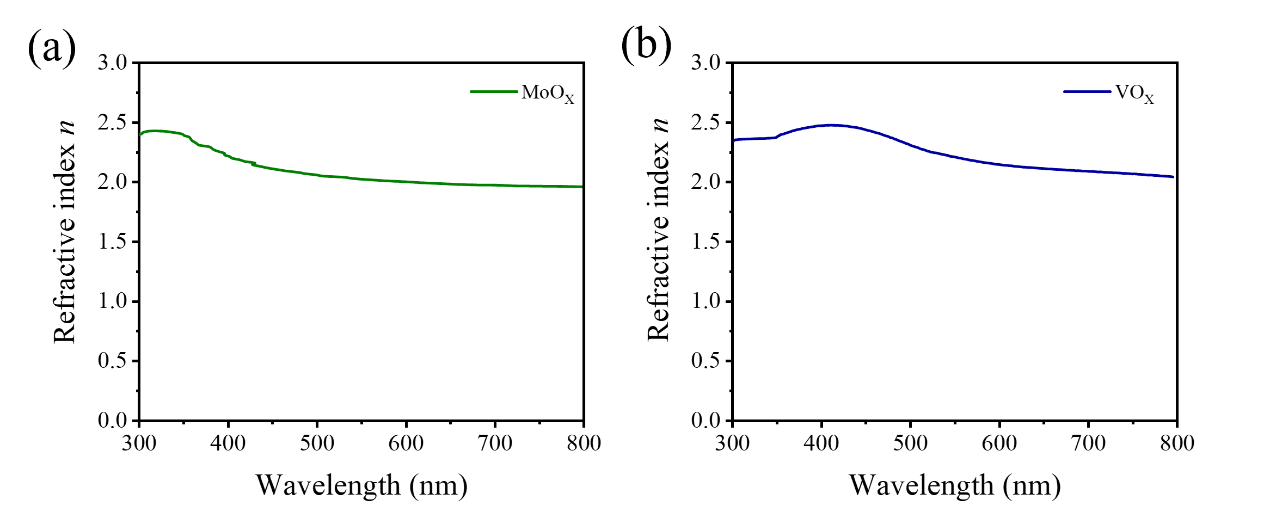
**Figure S3.** Dispersion of refractive index and extinction coefficient obtained from fitting of the spectroscopic ellipsometry data. ^[7]^

We have performed additional simulations to evaluate the MoO_X_ thickness, ranging from 2 nm to 7 nm (with 1 nm increments), while holding all other material parameters constant (see Tables S1-S3). The calculated V_OC_, J_SC_, FF and efficiency were shown in Figure S4. Device performance peaked at a MoO_X_ thickness of 4 nm, where optimal hole tunneling, stable V_OC_, and minimized series resistance collectively maximized efficiency.


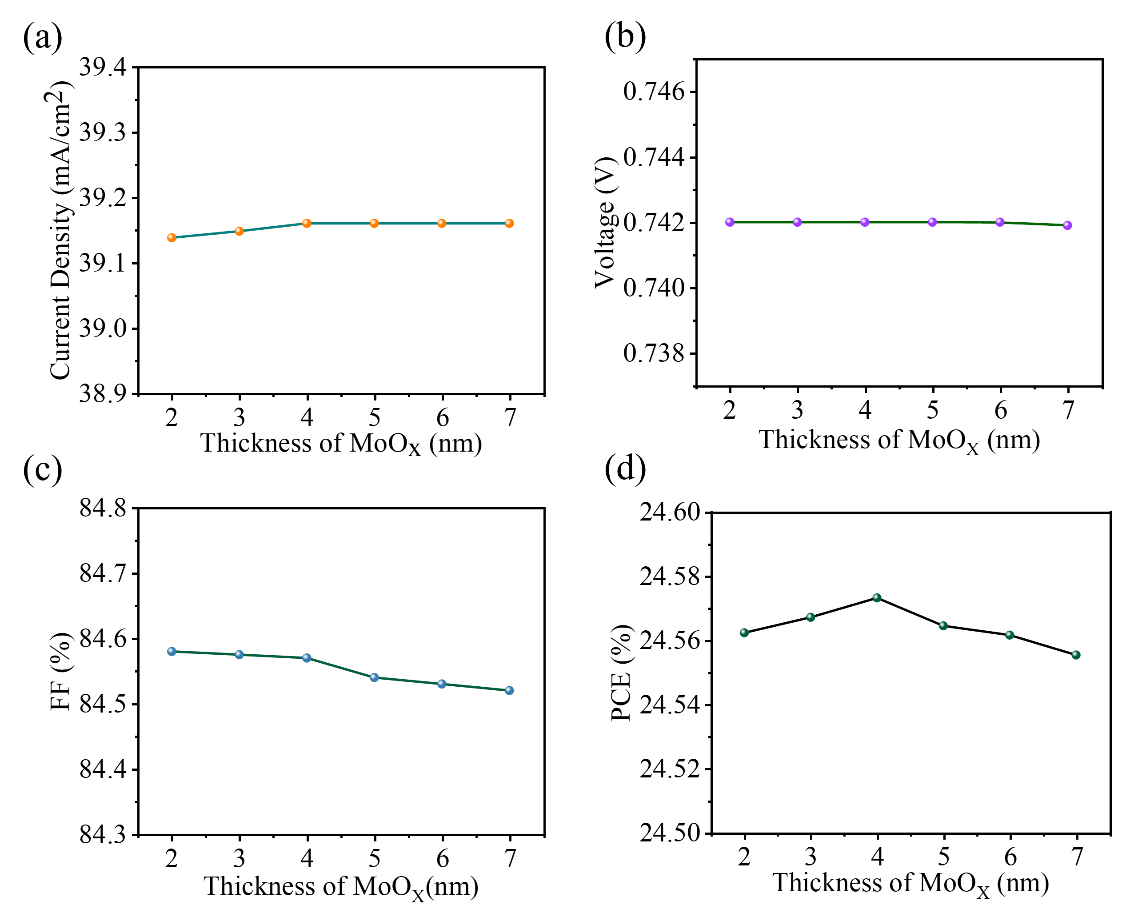


**Figure S4.** (a) J_SC_, (b) V_OC_, (c) FF, and (d) efficiency of silicon compound heterojunction solar cells, as a function of MoO_X_ thickness.

When taking into account the optical loss of the MoO_X_ hole transport layer on both backlit and illuminated sides, the EQE curve of MoO_X_ on the illuminated side outperformed that on the backlit side. This comparison demonstrated the advantages of placing MoO_X_ on the illuminated side, and enriched the overall understanding of the device. In addition, when MoO_X_ was on the illuminated side, its band gap is larger than *n*-a-Si: H (1.74eV^[8]^). This characteristic effectively mitigated the short-circuit current loss resulting from the absorption of doped amorphous silicon in the short-wave range, thereby improving the EQE response.

**Figure S5.** EQE of silicon compound heterojunction solar cells with MoO_X_ and *n*-a-Si:H on the illuminated side, respectively.

The valence band of *i*-a-Si:H aligned favorably with the conduction band of V:MoO_X_, enabling band-to-band tunneling for hole transport. This tunneling mechanism minimized energy loss and facilitates efficient carrier extraction.


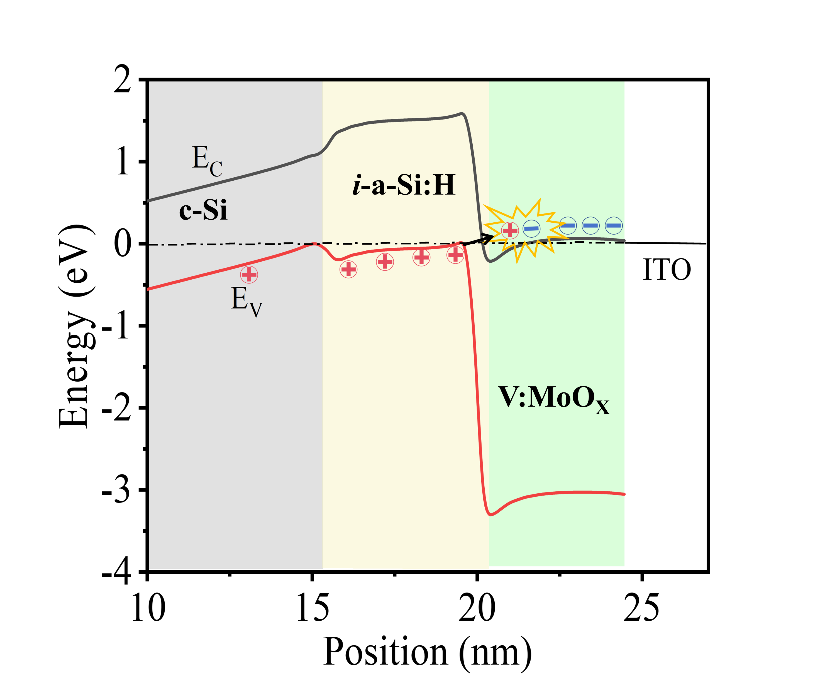


**Figure S6.** Band alignments of c-Si/*i*-a-Si:H/MoO_X_/ITO contact.

**Experimental validation**

We conducted experimental validation using V-doped MoO_X_ HTL devices, starting from commercially procured half-cells with structure of Ag-grid/ITO/*n-*a-Si:H/*i-*a-Si:H/c-Si/*i-*a-Si:H. Due to laboratory constraints, the following deviations from the simulated optimal design were unavoidable. (i) illumination configuration: the HTL (V:MoO_X_/ITO/Ag) was deposited on the rear side (*i-*a-Si:H side) via thermal evaporation, as our facility lacks screen-printing capabilities for front-side grid patterning. Consequently, illumination was applied through the *n-*a-Si:H side rather than HTL side. (ii) material synthesis limitation: The V:MoO_X_ layer was co-evaporated from a mixed powder of VO_X_ and MoO_X_ at a current parameter of 86.5 ~ 87 A with a deposition rate of 0.4 Ås^-1^, which may introduce inhomogeneity due to differing current parameters required for the evaporation of the precursors (MoO_X_ is around 65 ~ 66.5 A with a deposition rate of 0.1 Ås^-1^, and VO_X_ is around 85 ~ 92.5 A with a deposition rate of 0.1 Ås^-1^).

Despite these constraints, the results confirmed the efficacy of V-doping in MoO_X_. The V:MoO_X_ device achieved a PCE of 21.8%, outperforming the undoped MoO_X_ control (21.4%), with a significant V_OC_ increase from 0.695 V to 0.725 V, see Table S4 and Figure S4. The V_OC_ improvement aligned with our simulation-predicted suppression of oxygen vacancy (V_O_) induced non-radiative recombination in MoO_X_. Vanadium doping passivated V_O_ defects, reducing interfacial recombination V_OC_ losses. While the experimental PCE remained below the simulated 24.57% (front-illuminated V:MoO_X_ design), the trend confirmed the viability of V:MoO_X_ HTLs for silicon compound solar cells.

**
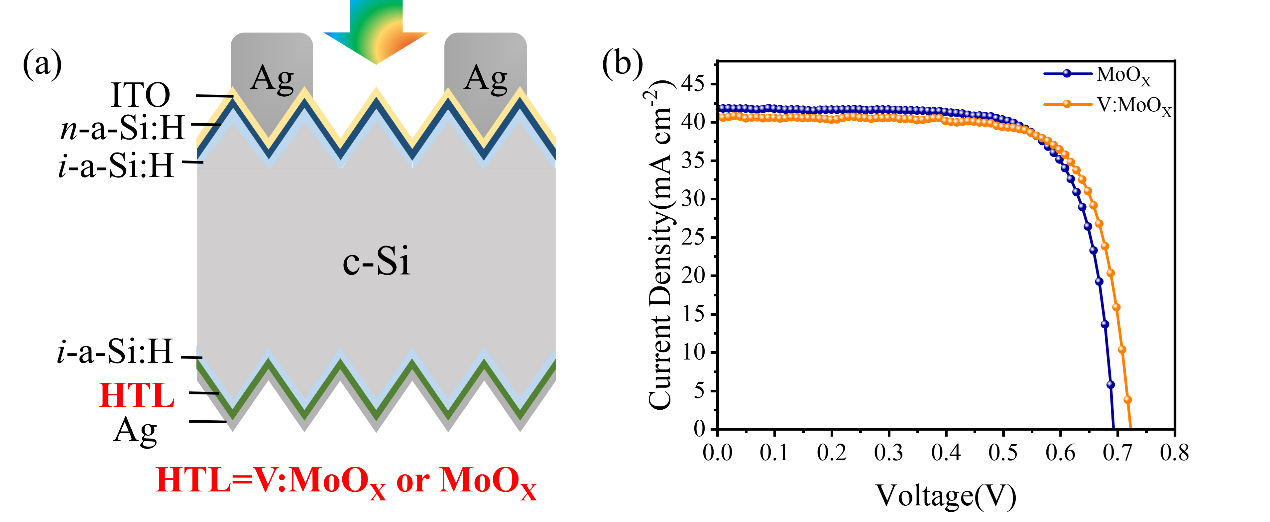
**

**Figure S7.** Current-voltage curves of silicon compound solar cells with hole transport layer of V:MoO_X_ and MoO_X_.

**Table S4.** Photovoltaic parameters (J_SC_, V_OC_, and FF) and efficiencies of silicon compound heterojunction solar cells with rear-side HTL of V:MoO_X_ or MoO_X_, respectively.

|  | V_OC_ (V) | J_SC_ (mA cm^-2^) | FF (%) | PCE (%) |
| --- | --- | --- | --- | --- |
| MoO_X_ | 0.695 | 41.8 | 73.6 | 21.4 |
| V:MoO_X_ | 0.725 | 40.7 | 74.0 | 21.8 |

**Device fabrication**

The V-doped MoO_X_ HTL was fabricated via thermal evaporation on commercially procured half-cell substrates with an initial structure of Ag-grid/ITO/*n-*a-Si:H/*i-*a-Si:H/c-Si/*i-*a-Si:H. Prior to deposition, the rear surfaces of substrates were cleaned by 4 wt% hydrofluoric acid (HF) solution for native oxide removal, thorough rinsing with deionized water, and drying under a nitrogen (N_2_) stream to minimize surface contamination. The HTL film was thermally evaporated from the mixed powder of MoO_3_ and V_2_O_5_. During the deposition process, the evaporation rate was maintained at 0.4 Ås^-1^, and the vacuum level was below 4×10^-4^ Pa. The thickness of the film was monitored by a quartz crystal film thickness instrument and calibrated by ellipsometry. This ensured the precision and uniformity of the HTL film. The thermal evaporation equipment features a real-time adjustment system capable of dynamically regulating the current, maintaining consistent evaporation rates and film thicknesses, thereby ensuring the high-quality stability of each batch of films. Finally, a 200-nm-thick silver back electrode was deposited via room-temperature thermal evaporation to complete the device structure.

**Reference**

[1] J. Li, Q. Kang, Y. Wang, Z. Zhou, Z. Sun, H. Zhang, W. Lu, X. Tao, S. T. Zhang, X. Chen, Z. Zheng, H. Yan, D. Li, Y. Zhang, Advanced Functional Materials 2023, 34, 2310619.

[2] M. Q. Khokhar, K. Mallem, X. Fan, Y. Kim, S. Q. Hussain, E.-C. Cho, J. Yi, ECS Journal of Solid State Science Technology 2022, 11, 085001.

[3] H. Lin, M. Yang, X. Ru, G. Wang, S. Yin, F. Peng, C. Hong, M. Qu, J. Lu, L. Fang, C. Han, P. Procel, O. Isabella, P. Gao, Z. Li, X. Xu, Nature Energy 2023, 8, 789.

[4] A. Ait Abdelkadir, E. Oublal, M. Sahal, B. M. Soucase, A. Kotri, M. Hangoure, N. Kumar, Silicon 2022, 15, 2125.

[5] L. Bai, Y. Zhang, L. Zhang, Y. Zhang, L. Sun, N. Ji, X. Li, H. Si, Y. Zhang, H. Huang, Nano energy 2018, 53, 982.

[6] P.-R. Huang, Y. He, C. Cao, Z.-H. Lu, Scientific Reports 2014, 4, 7131.

[7] a) Y. Jiang, S. Cao, L. Lu, G. Du, Y. Lin, J. Wang, L. Yang, W. Zhu, D. Li, Nanoscale Research Letters 2021, 16, 87; b) Q. He, X. Xu, Y. Gu, M. Wang, J. Yao, Y. Jiang, M. Sun, T. Ao, Y. Lian, F. Wang, X. Li, Journal of Physics D: Applied Physics 2016, 49, 400105.

[8] S. Mandal, S. Dhar, G. Das, S. Mukhopadhyay, A. K. Barua, Solar Energy 2016, 124, 278.
